# Associations between biomarkers of cellular senescence and physical function in humans: observations from the lifestyle interventions for elders (LIFE) study

**DOI:** 10.1007/s11357-022-00685-2

**Published:** 2022-11-11

**Authors:** Roger A. Fielding, Elizabeth J. Atkinson, Zaira Aversa, Thomas A. White, Amanda A. Heeren, Sara J. Achenbach, Michelle M. Mielke, Steven R. Cummings, Marco Pahor, Christiaan Leeuwenburgh, Nathan K. LeBrasseur

**Affiliations:** 1 Nutrition, Exercise Physiology and Sarcopenia Laboratory, Jean Mayer USDA Human Nutrition Research Center On Aging, Tufts University, Boston, MA USA; 2 Department of Quantitative Health Sciences, Mayo Clinic, Rochester, MN USA; 3 Robert and Arlene Kogod Center On Aging, Mayo Clinic, 200 First Street SW, Rochester, MN 55905 USA; 4 Department of Physical Medicine and Rehabilitation, Mayo Clinic, Rochester, MN USA; 5 Department of Epidemiology and Prevention, Wake Forest University School of Medicine, Winston-Salem, NC USA; 6 Departments of Medicine, Epidemiology and Biostatistics, University of California San Francisco, San Francisco, CA USA; 7 Research Institute, California Pacific Medical Center, San Francisco, CA USA; 8 Institute On Aging, University of Florida, Gainesville, FL USA

**Keywords:** Sarcopenia, Frailty, Biomarkers, Physical function, Aging, Short physical performance battery

## Abstract

Cellular senescence is a plausible mediator of age-associated declines in physical performance. To test this premise, we examined cross-sectional associations between circulating components of the senescence-associated secretory phenotype (SASP) and measures of physical function and muscle strength in 1377 older adults. We showed significant associations between multiple SASP proteins and the short physical performance battery (SPPB), its subcomponents (gait speed, balance, chair rise time), and 400-m walk time. Activin A, ICAM1, MMP7, VEGFA, and eotaxin showed strong associations based on gradient boost machine learning (GBM), and, when combined with other proteins, effectively identified participants at the greatest risk for mobility disability (SPPB score $\le$ 7). Senescence biomarkers were also associated with lower grip strength, and GBM identified PARC, ADAMTS13, and RANTES as top candidates in females, and MMP2, SOST, and MCP1 in males. These findings highlight an association between senescence biomarkers and physical performance in older adults. ClinicalTrials.gov Identifier: NCT01072500.

**Supplementary Information:**

The online version contains supplementary material available at 10.1007/s11357-022-00685-2.

## Introduction

Advanced age is reflected by deficits in mobility and strength across species [1]. In humans, slow gait speed, poor endurance, and muscle weakness amplify risk for adverse clinical outcomes, including falls, fractures, institutionalization, and mortality [2–5]. The universal and progressive nature of functional decline suggests it may be driven by fundamental mechanisms of aging.

Cellular senescence is a hallmark of aging [6, 7]. Senescent cells accumulate in most tissues with advancing age in response to diverse forms of genotoxic, proteotoxic, metabolic, and inflammatory stress and are characterized by distinct changes in morphology, upregulation of cell cycle regulators and anti-apoptosis pathways, alterations in metabolism and, notably, a robust and heterogenous senescence-associated secretory phenotype (SASP) [8]. Through the SASP, comprised of cytokines, chemokines, matrix remodeling proteins, growth factors, and a host of other bioactive molecules, senescent cells mediate age-associated tissue inflammation, deterioration, and remodeling, and, simultaneously, compromise regeneration both locally and systemically [9]. Interestingly, in preclinical models, enrichment of the SASP through senescent cell transplantation compromises muscle performance and physical function [10], while pharmacological suppression of the SASP has been shown to improve these parameters [11, 12].

In addition to mediating the systemic effects of senescent cells, circulating concentrations of SASP proteins have been used as biomarkers of senescent cell burden. Correspondingly, senescence biomarkers have been associated with chronological age and clinical manifestations of advanced biological age (i.e., disease burden and adverse surgical outcomes) in humans [13, 14]. The extent to which senescence biomarkers associate with measures of mobility and strength in older adults, to our knowledge, has not yet been investigated. However, based on the above, these data would lend critical support to the premise that targeted elimination of senescent cells is a viable opportunity to extend human healthspan.

To address this gap, we used baseline data and biospecimens obtained from older females and males with mobility limitations who participated in the Lifestyle Interventions in Elders (LIFE) study, a large, single-blind, randomized clinical trial conducted at eight centers across the USA [15]. The plasma concentrations of 27 proteins we previously identified as candidate senescence biomarkers were measured using multiplexed bead-based immunoassays or an enzyme-linked immunosorbent assay (ELISA) [13]. Physical function was assessed by the Short Physical Performance Battery (SPPB), which is a composite measure of gait, balance, and sit-to-stand ability, and the 400-m (400 m) walk test, a measure of mobility and endurance. Importantly, poor performance on the SPPB and 400 m walk is associated with a heightened risk of disability, institutionalization, and mortality among older adults [2, 3, 16, 17]. Isometric handgrip strength was used as a measure of whole-body strength. It has been widely ascertained in large epidemiological cohort studies worldwide and is associated with important health outcomes including falls, fractures, and mortality [4, 18–20]. In this study, we tested the overall hypothesis that higher plasma biomarkers of cellular senescence would be associated with poorer performance on these objective and clinically important measures of physical function and muscle strength.

## Methods

### Design

The LIFE study was a phase 3 multi-center randomized controlled trial of a group-based physical activity program (moderate intensity, walking plus lower extremity strength training) compared to a “successful aging” health education program which was conducted from February 2010 to December 2013, enrolling 1635 sedentary older adults [15]. Participants were recruited across eight centers in the USA (University of Florida, Gainesville and Jacksonville, Florida; Northwestern University, Chicago, Illinois; Pennington Biomedical Research Center, Baton Rouge, Louisiana; University of Pittsburgh, Pittsburgh, Pennsylvania; Stanford University, Stanford, California; Tufts University, Boston, Massachusetts; Wake Forest School of Medicine, Winston-Salem, North Carolina; and Yale University, New Haven, Connecticut). Females and males aged 70–89 years were recruited with the following characteristics: (1) sedentary lifestyle (defined as reporting < 20 min/day of regular physical activity and < 125 min/week of moderate physical activity; (2) at high risk of disability based upon a score between 4 and 10 on the SPPB; (3) ability to complete the 400 m walk test without an assistive device within 15 min; (4) absence of cognitive impairment; and (5) willingness to consent to randomization. Study exclusions were unstable chronic disease and factors that would likely affect adherence to the intervention or underlying conditions that might limit survival. Institutional Review Board (IRB) approval and informed consent were obtained at the study centers. The present analyses were conducted on coded data with no personal identifiers and deemed exempt from the requirement for IRB approval (45 CFR 46.101, item 4).

### Performance outcome measures

Study outcomes of interest were obtained at baseline and included the SPPB (comprised of 4 m gait speed, repeated chair rise time, and measures of standing balance), 400 m walk test, and isometric hand grip strength. Detailed descriptions, performance characteristics, and quality control procedures are provided elsewhere [15].

### Measurement of circulating senescence biomarkers

During the trial, plasma specimens were collected at the baseline, 6-, 12- and 24-month visits, and immediately stored at − 80 ℃. For the current study, archived specimens were requested and shipped from the National Institute of Aging (NIA) Aging Research Biobank to Mayo Clinic for analysis.

The concentrations of protein biomarkers in plasma samples were quantified using commercially available multiplex magnetic bead-based immunoassays (R&D Systems) on the Luminex xMAP multianalyte profiling platform and analyzed on MAGPIX System (Merck Millipore). All assays were performed according to the manufacturer’s protocols. The biomarkers quantified with this method included ADAMTS13, eotaxin, Fas, GDF15, ICAM1, IL6, IL7, IL8, IL10, IL15, MCP1, MDC, MMP1, MMP7, MMP9, MPO, OPN, PAI1, PARC, RAGE, RANTES, SOST, TNFR1, TNFR2, TNFα, and VEGFA. Activin A concentration was determined by a Quantikine ELISA Kit (R&D Systems) according to the manufacturer’s instructions.

Assay performance characteristics are reported in Supplemental Table 10. In cases where a biomarker was below the limit of detection in a sample, a value of half of the lowest measured value for that analyte was assigned.

### Data analysis

Continuous variables were summarized using mean (SD) and compared using the Wilcoxon rank sum. Variables were compared pairwise using unadjusted and adjusted Spearman correlations. False discovery rate “*q*-values” were calculated for the correlation tables to help account for multiple testing. Biomarkers were evaluated for skewed distributions and if appropriate were log-transformed. Then all biomarkers were standardized by subtracting the mean and dividing by the standard deviation, then K-nearest neighbor imputation was applied to impute the small number of missing values. Prior to modeling the data was split into a training and testing set. Gradient boosting machine learning using the gbm package in R was used to create a predictive model for each endpoint [21]. Model hyperparameters (depth of the tree, number of trees, and size of the shrinkage parameter) were determined using cross-validation using the training dataset. Models were fit using (1) all the variables, (2) the top 10 biomarkers plus age, sex, race, and body mass index (BMI), and 3) age, sex, race, and bmi. The area under the receiver operator curves was calculated by applying pre-established cutoffs to the predicted values from the models. All analyses were conducted using R version 4.0.3 [22].

### Data availability

LIFE study datasets are available through the NIA Aging Research Biobank, https://agingresearchbiobank.nia.nih.gov/studies/life/. Biomarker data presented in the current study will be made available by the investigative team upon reasonable request.

## Results

### Demographic and clinical characteristics of participants

We studied 1377 of the 1635 LIFE study participants with baseline blood samples and clinical data available for analysis. Similar to the overall cohort, the participants herein were predominantly female (66%) with a mean age of 79.0 years; 77% were White and 16% were Black (Table 1). Prevalent comorbid conditions included high blood pressure (71%), diabetes (23%), chronic pulmonary disease (15%), and cancer (22%); 20% of participants were classified as frail, based on the study of osteoporotic fractures index [23].

**Table 1:** Demographic and clinical characteristics and physical function measures in study participants. Data reported as mean (standard deviation) unless otherwise noted

	**Females (*N* = 911)**	Males (*N* = 466)	All (*n* = 1377)
Age, years	78.64 (5.23)	79.28 (5.20)	78.86 (5.22)
Race and ethnicity, *n* (%)			
White	660 (72.5%)	403 (86.9%)	1063 (77.4%)
Black or African American	187 (20.5%)	39 (8.4%)	226 (16.4%)
Hispanic	40 (4.4%)	10 (2.2%)	50 (3.6%)
Asian	10 (1.1%)	4 (0.9%)	14 (1.0%)
Other	13 (1.4%)	8 (1.7%)	21 (1.5%)
Missing	1	2	3
Body mass index, kg/m^2^	30.33 (6.18)	29.97 (5.40)	30.21 (5.93)
Health conditions, *n* (%)			
High blood pressure	650 (72%)	325 (71%)	975 (71%)
Diabetes	208 (23%)	139 (30%)	347 (25%)
Cancer	171 (19%)	136 (29%)	307 (22%)
Chronic lung disease	149 (16%)	56 (12%)	205 (15%)
MI/ heart attack	54 (6%)	52 (11%)	106 (8%)
Stroke	62 (7%)	34 (7%)	96 (7%)
Physical function			
SPPB score (0–12)	7.3 (1.6)	7.6 (1.6)	7.4 (1.6)
400 m walk, s	516 (108)	484 (114)	505 (111)
Gait speed, m/sec	0.75 (0.16)	0.80 (0.16)	0.77 (0.16)
Repeated chair stand, s	16.86 (4.89)	16.69 (6.38)	16.80 (5.44)
Grip strength, kg	20.51 (6.41)	32.79 (9.46)	24.76 (9.59)
Frailty, *n* (%)	192 (21%)	76 (16%)	268 (20%)

### Senescence biomarkers in LIFE study participants

We examined the plasma concentrations of 27 proteins that were previously identified as components of the SASP that were elicited by several human senescent cell types and associated with chronological age and/or multimorbidity (Supplemental Table 1)[13]. In the present study, activin A, GDF15, RAGE (all *r* > 0.2 and *P* < 0.001), and 10 other biomarkers demonstrated significant positive associations with chronological age (Supplemental Table 2). Levels of MDC, PAI1, and RANTES were higher in females compared to males (Table 2), while eotaxin, GDF15, ICAM1, IL6, osteopontin (OPN), SOST, TNFR1, and TNFR2 were higher in males.

**Table 2:** Biomarker concentrations (pg/mL) in study participants. Data reported as mean (standard deviation)

	Females (*N* = 911)	Males (*N* = 466)	*P* value^1^	*Q* value^2^
Activin A	400.1 (166.0)	389.3 (149.7)	0.395	0.627
ADAMTS13	1,213,626 (486,866)	1,224,958 (449,265)	0.652	0.787
Eotaxin	**120.4 (62.5)**	**134.1 (81.0)**	**0.001**	**0.002**
Fas	7556 (2,548)	7794 (3,225)	0.057	0.118
GDF15	**1475 (1063)**	**1852 (1126)**	** < 0.001**	** < 0.001**
ICAM1	**314,849 (292,101)**	**418,690 (393,418)**	** < 0.001**	** < 0.001**
IL6	**1.66 (3.66)**	**1.79 (3.52)**	**0.039**	0.096
IL7	2.78 (2.84)	2.78 (3.46)	0.056	0.118
IL8	4.73 (6.11)	4.57 (5.51)	0.788	0.818
IL15	1.59 (1.08)	1.60 (1.44)	0.729	0.787
MCP1	184.0 (90.1)	188.1 (100.1)	0.438	0.657
MDC	**604.4 (281.0)**	**511.1 (223.6)**	** < 0.001**	** < 0.001**
MMP1	874.9 (836.7)	957.3 (1060.5)	0.683	0.787
MMP2	361,325 (100,211)	362,558 (92,608)	0.588	0.756
MMP7	2902 (1,568)	2881 (1,714)	0.524	0.745
MMP9	45,377 (26,520)	46,841 (29,700)	0.962	0.962
MPO	39,239 (38,647)	36,513 (26,959)	0.293	0.495
OPN	**28,435 (14,924)**	**31,845 (16,373)**	** < 0.001**	** < 0.001**
PAI1	**27,243 (20,390)**	**23,897 (19,453)**	** < 0.001**	** < 0.001**
PARC	69,117 (36,021)	71,686 (63,469)	0.555	0.750
RAGE	2219 (1,070)	2297 (1,138)	0.181	0.326
RANTES	**36,949 (50,619)**	**31,003 (45,312)**	** < 0.001**	**0.001**
SOST	**504.5 (251.9)**	**657.3 (352.0)**	** < 0.001**	** < 0.001**
TNFα	4.58 (4.43)	4.80 (4.15)	0.174	0.326
TNFR1	**1507 (614)**	**1637 (653)**	** < 0.001**	** < 0.001**
TNFR2	**3505 (1775)**	**3,785 (1792)**	** < 0.001**	** < 0.001**
VEGFA	83.84 (47.02)	83.29 (44.24)	0.713	0.787

^1^Wilcoxon rank sum test; ^2^Benjamini and Hochberg false discovery rate

Bold font indicates a significant difference between females and males

### Association between senescence biomarkers and measures of physical function

#### SPPB

**Fig. 1:**
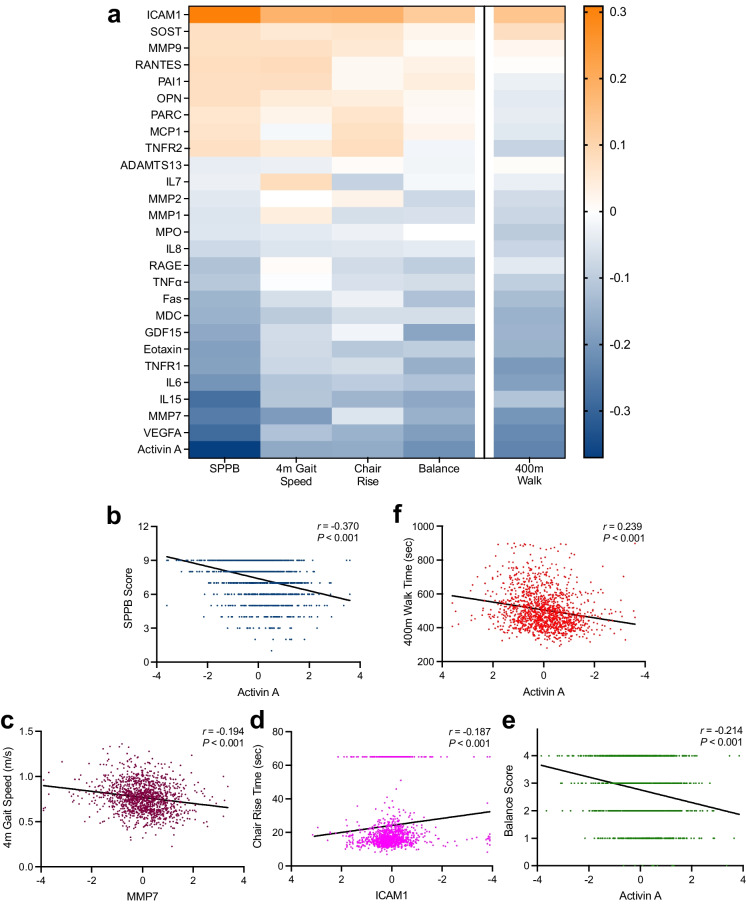
Biomarkers of cellular senescence associated with measures of physical functioning. **a** Heatmap representing the unadjusted Spearman’s rank correlations of 27 senescence-associated biomarkers and physical function outcomes (SPPB, 4 m gait speed, chair rise time, balance score, and 400 m walk time) (*n* = 1377). **b** Unadjusted Spearman correlation between SPPB score (0–12 score) and activin A, with Spearman Rho and *P* value in top right. **c** Unadjusted Spearman correlation between 4 m gait speed (m/s) and MMP7, with Spearman Rho and *P* value in top right. **d** Unadjusted Spearman correlation between Chair Rise Time (s) and ICAM1, with Spearman Rho and *P* value in top right. **e** Unadjusted Spearman correlation between balance score (0–4 score) and activin A, with Spearman Rho and *P* value in top right. **f** Unadjusted Spearman correlation between 400 m walk time (s) and activin A, with Spearman Rho and *P* value in top right. All biomarker measures were standardized by subtracting the mean and dividing by the standard deviation

We first evaluated whether circulating SASP protein concentrations were associated with the composite SPPB score. Unadjusted Spearman rank correlation coefficients demonstrated significant inverse associations (i.e., higher protein level, poorer performance) between 13 proteins and SPPB score, with activin A (*r* =  − 0.37, *P* < 0.001), VEGFA (*r* =  − 0.29, *P* < 0.001), and IL15 (*r* =  − 0.28, *P* < 0.001) being most strongly associated (Fig. 1, Supplemental Table 3). ICAM1 (*r* = 0.31, *P* < 0.001) was positively associated with SPPB. The associations between biomarkers and SPPB remained significant after adjusting for age, sex, race, and BMI and multiple comparisons.

**Fig. 2:**
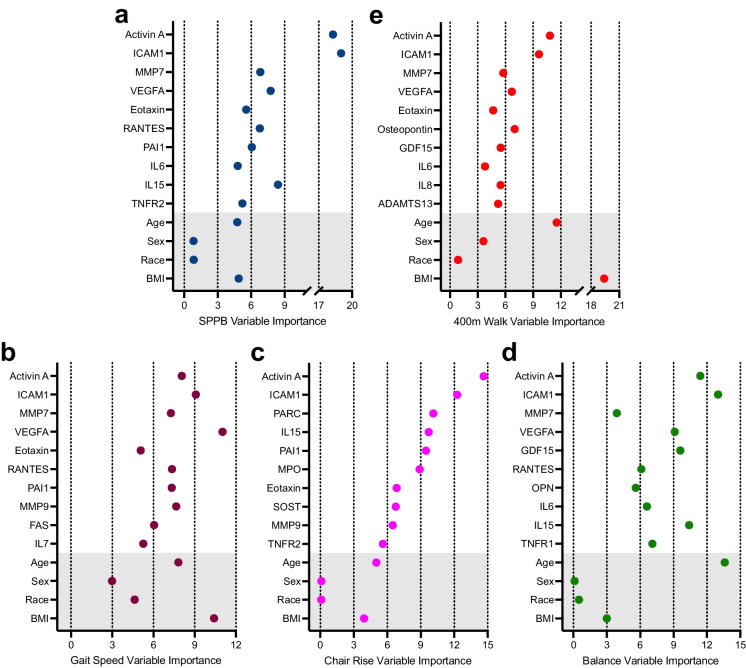
Biomarkers of senescence are robust determinants of physical functioning. The top ten biomarkers along with age (years), sex (female, male), race (White, Black or African American, Hispanic, Asian, or Other), and BMI (kg/ht m.^2^) selected by gradient boost modeling for **a** SPPB (0–12 score). **b** 4 m gait speed (m/s), **c** chair rise time (s), **d** balance score (0–4 score), and **e** 400 m walk time

**Fig. 3:**
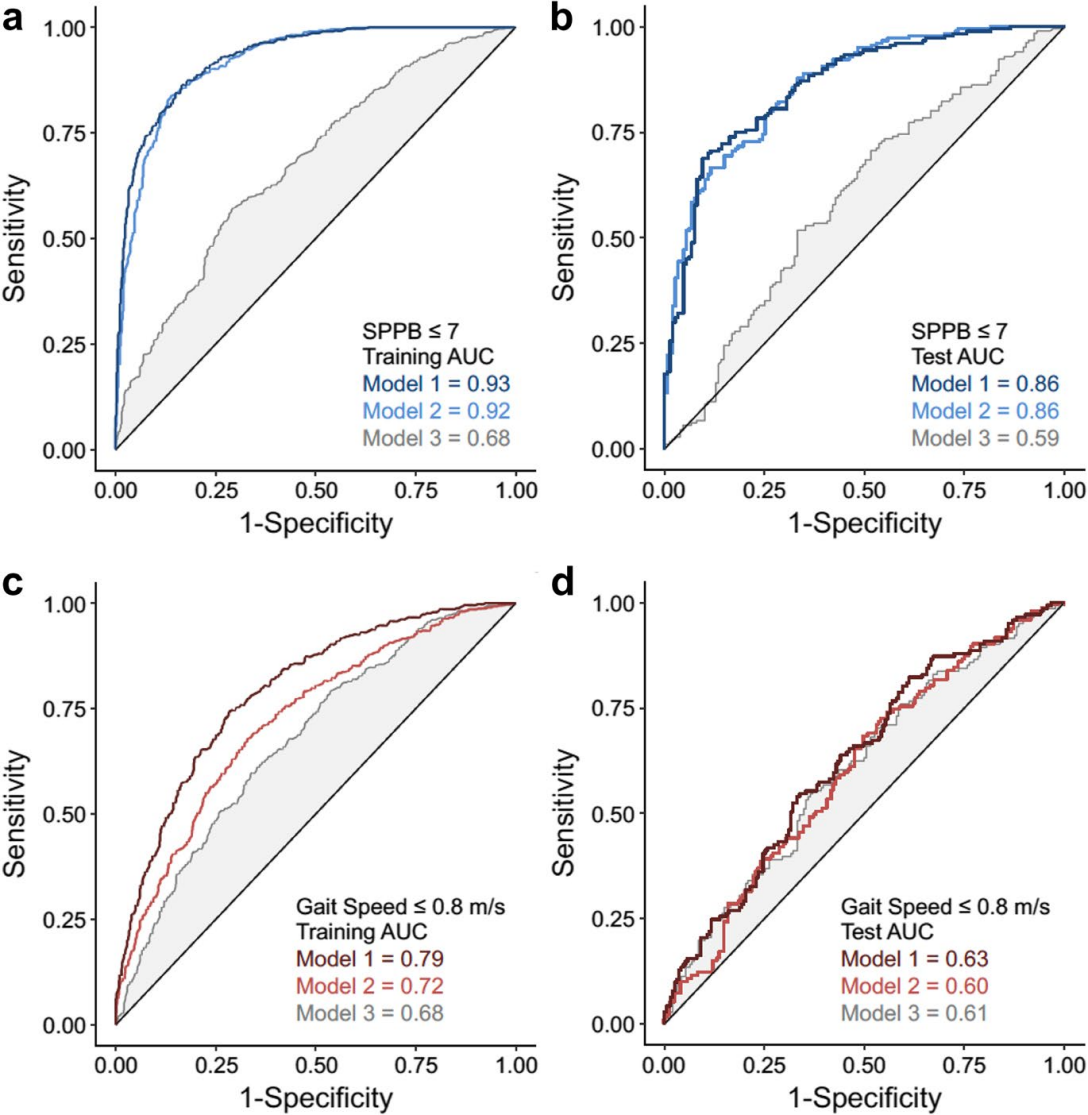
Biomarkers of cellular senescence identify risk for mobility disability. Receiver operator characteristic (ROC) curves to distinguish participants with higher (8–12) and lower SPPB scores (< 8) in **a** training data (*n* = 1,050) and **b** test data (*n* = 327). ROC curve to distinguish between participants with slower (≤ 0.8 m/s) and faster gait speeds, **c** in training data (*n* = 1049) and **d** test data (*n* = 327). Figures reflect the discriminatory ability of the top ten biomarkers selected by gradient boost modeling plus age, sex race, and BMI (Model 1), the top ten biomarkers alone (Model 2), and age, sex, race, and BMI alone (Model 3). The area under the curve values appears in the bottom right of each panel

The concentrations of activin A, ICAM1, MMP7, VEGFA, eotaxin, RANTES, PAI1, IL6, IL15, and TNFR2 were identified as the 10 most important predictors of SPPB score using gradient boost machine learning (GBM) regression modeling (Fig. 2a). In general, senescence biomarkers had a greater importance than age, BMI, sex, and race. We next examined the ability of the top ten biomarkers to identify participants classified as having the greatest risk for mobility disability, defined by an SPPB score $\le$ 7 [24], using the receiver operator characteristic area under the curve (ROC AUC). Impressively, the ROC AUC of the top 10 biomarkers plus age, sex, race, and BMI was 0.93 for the training set (*n* = 1,050) and 0.86 for the test set (*n* = 327) (Fig. 3a). In comparison, the ROC AUC of age, sex, race, and BMI for SPPB score $\le$ 7 was 0.68 for the training set and 0.59 for the test set, suggesting the biomarkers have higher predictive power for risk for mobility disability relative to age, sex, race, and BMI.

Comparison of the associations between senescence-associated biomarkers and the individual components of the SPPB score (4 m gait speed, chair rise time, and standing balance score) revealed remarkable consistency. In particular, higher concentrations of activin A, VEGFA, IL15, IL6, and eotaxin were significantly associated with poorer performance, and ICAM1 better performance, across all three measures even after adjusting for age, sex, race, and BMI and multiple comparisons (Fig. 1, Supplemental Tables 4, 5, and 6). Moreover, GBM analysis identified activin A and ICAM1 among the top 10 predictors of 4 m gait speed, chair rise time, and standing balance score, and MMP7, VEGFA, eotaxin, RANTES, PAI1, MMP9, as top predictors of two of the three measures of physical performance (Fig. 2b-d).

Given the independent predictive ability of slow gait speed on a multitude of clinical outcomes in older adults including hospitalization, institutionalization, and mortality [5, 25–27], we assessed the ability of the top 10 biomarkers to distinguish between participants with and without gait speeds $\le$ 0.8 m/s. The ROC AUC of the top 10 biomarkers plus age, sex, race, and BMI was 0.79 for the training set and 0.63 for the test set (*n* = 327) (Fig. 3b). In comparison, the ROC AUC of age, sex, race, and BMI for SPPB score $\le$ 0.8 m/s was 0.68 for the training set and 0.61 for the test set, suggesting the biomarkers have moderately better predictive power for clinically significant slow gait speed relative to age, sex, race, and BMI.

#### 400 m walk

We next investigated whether circulating concentrations of SASP proteins were related to the time to complete the 400 m walk. Unadjusted Spearman rank correlation coefficients demonstrated significant associations between 18 biomarkers and 400 m walk time (Fig. 1, Supplemental Table 7). Activin A (*r* = 0.24, *P* < 0.001), VEGFA (*r* = 0.23, *P* < 0.001), MMP7 (*r* = 0.21, *P* < 0.001), and TNFR1 (*r* = 0.20, *P* < 0.001) exhibited the strongest positive associations (higher protein concentrations, longer time to complete walk) and ICAM1 the strongest of two biomarkers with negative associations (*r* =  − 0.14, *P* < 0.001). Except for MMP2 and SOST, the associations between biomarkers and 400 m walk time remained significant after adjustment for age, sex, race, and BMI and multiple comparisons.

Notably, further analysis of the relationship between senescence-associated proteins and 400 m walk time by GBM revealed that the top five of the 10 strongest predictors were the same as those identified for the SPPB; namely, activin A, ICAM1, MMP7, VEGFA, and eotaxin. IL6 was also in common, while OPN, GDF15, IL8, and ADAMTS13 were included only in the top 10 of predictors of 400 m walk time (Fig. 2e). In contrast to the SPPB, age and BMI were strong determinants of 400 m walk time.

### Association between senescence biomarkers and muscle strength

We next evaluated the extent to which components of the SASP were related to the grip strength of the dominant hand. Because of the marked (60%) difference in strength between sexes, data from females and males were analyzed separately.

**Fig. 4:**
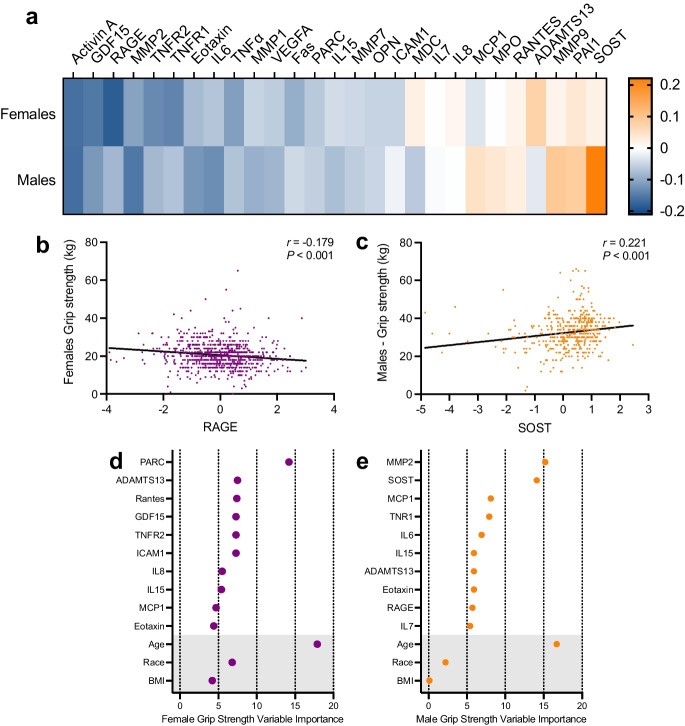
Biomarkers of cellular senescence and muscle strength. **a** Heatmap representing the unadjusted Spearman’s rank correlations of 27 senescence-associated biomarkers and grip strength in females (top row) and males (bottom row). **b** Unadjusted Spearman correlation between grip strength (kg) in females and RAGE, with Spearman Rho and *P* value in top right. c, Unadjusted Spearman correlation between grip strength (kg) in males and SOST, with Spearman Rho and *P* value in the top right. All biomarker measures were standardized by subtracting the mean and dividing by the standard deviation. Top ten biomarkers along with age (years), sex (female, male), race (White, Black or African American, Hispanic, Asian, or Other)), and BMI (kg/ht m.^2^) selected by gradient boost modeling for grip strength in females (**d**) and males (**e**)

Among females, unadjusted Spearman rank correlations revealed that ten biomarkers were significantly associated with maximum hand grip strength (Fig. 4a, Supplementary Table 8). Of the nine biomarkers exhibiting negative associations (higher protein level, lower strength), RAGE (*r* = -0.18, *P* < 0.001), activin A (*r* = -0.16, *P* < 0.001), GDF15 (*r* = -0.15, *P* < 0.001), and TNFR1 (*r* = -0.14, *P* < 0.001) were strongest and remained significant after adjustment for covariates and multiple comparisons. ADAMTS13 had a positive, albeit weak, association with grip strength (*r* = 0.08, *P* = 0.028) that was no longer significant after adjustment for age, race, and BMI. Among males, activin A (*r* =  − 0.16, *P* = 0.001), MMP2 (*r* =  − 0.15, *P* = 0.002), and IL6 (*r* =  − 0.12, *P* = 0.008) demonstrated negative associations with maximum grip strength that remained significant after adjustment, while SOST was positively associated (*r* = 0.22, *P* < 0.001), (Fig. 4a, Supplementary Table 9).

The GBM regression model for maximum grip strength also highlighted sex differences. Only circulating concentrations of ADAMTS13, IL15, and eotaxin were in common between the top 10 variables for females and males (Fig. 4d and 4e). PARC was the strongest biomarker in females as was MMP2 in males. Age was identified as a variable of high importance for grip strength in both sexes.

**Fig. 5:**
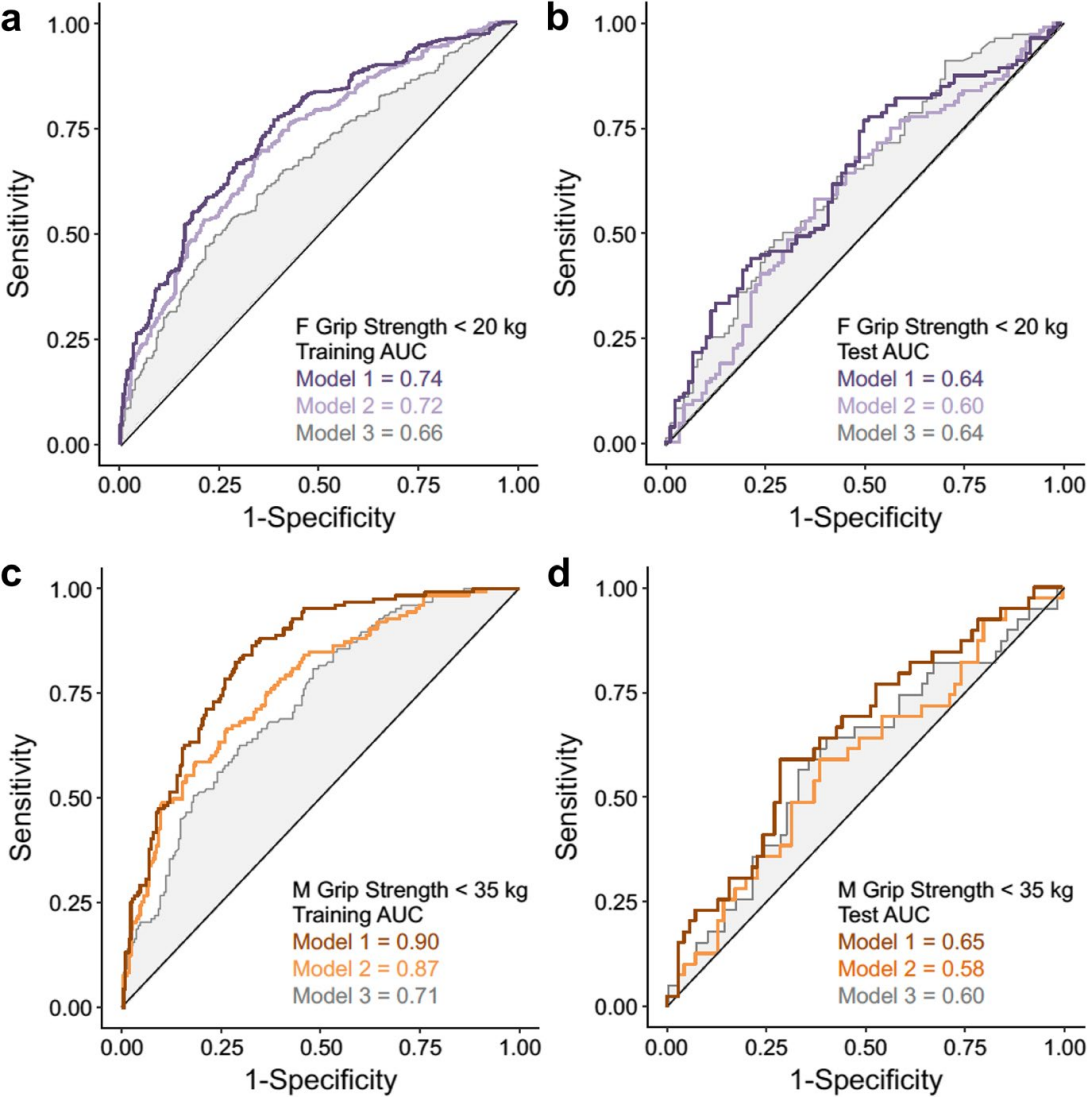
Biomarkers of cellular senescence and risk for muscle weakness. Receiver operator characteristic (ROC) curves to identify female participants with poor grip strength (< 20 kg) in **a** training data (*n* = 649) and **b** test data (*n* = 331). ROC curves to identify male participants with poor grip strength (< 35.5 kg) in **c** training data (*n* = 331) and **d** test data (*n* = 109). Figures reflect the discriminatory ability of the top ten biomarkers selected by gradient boost modeling plus age, sex race, and BMI (Model 1), the top ten biomarkers alone (Model 2), and age, sex, race, and BMI alone (Model 3). The area under the curve values appears in the bottom right of each panel

The Sarcopenia Definitions and Outcomes Consortium identified < 20.0 kg in females and < 35.5 kg in males as cut-points for low maximum grip strength (i.e., muscle weakness), which is associated with increased risk for mobility limitations and mortality in both sexes [5, 28]. The ROC AUC of the top 10 biomarkers plus age, race, and BMI was 0.74 for the training set in females (*n* = 649) and 0.90 in males (*n* = 331), and 0.64 for the test set in females (*n* = 331) and 0.65 in males (n = 109) (Fig. 5a-d). In comparison, the ROC AUC of age, race, and BMI for muscle weakness was 0.66 for females and 0.71 for males in the training set, and 0.64 for females and 0.60 for males in the test set. The test data suggest the biomarkers are comparable to age, race, and BMI for predicting muscle weakness. In addition, we applied more restrictive grip strength cut-points (females < 16.0 kg; males < 27.0 kg) that have been recommended by the European Working Group on Sarcopenia in Older People (EWGSOP) [29]. Using these criteria, the results were similar with ROC AUC of the top 10 biomarkers plus age, race, and BMI being 0.76 for the training set in females and 0.95 in males, and 0.65 for the test set in females and 0.67 in males. In comparison, the ROC AUC of age, race, and BMI for muscle weakness was 0.74 for females and 0.67 for males in the training set and 0.58 for females and 0.60 for males in the test set.

## Discussion

Despite the prevalence and the major impact of age-associated declines in muscle performance and physical function on independence and quality of life, their causes remain poorly understood. Here, in a large cohort of community-dwelling older adults, we identified significant associations between biomarkers of cellular senescence, a fundamental mechanism of aging, and rigorously collected measures of strength and mobility. Our data support the premise that senescent cells and the SASP may contribute to weakness and functional limitations in older adults and highlight the potential for interventions that target this hallmark of aging to optimize late-life health.

The SPPB is a highly informative composite measure of physical functioning. Intuitively, as a composite measure, the SPPB reflects the health of multiple, integrated physiological systems, e.g., musculoskeletal, cardiovascular, and neurological. This may partly account for the large number of circulating SASP proteins associated with the SPPB and, when used in combination, the impressive sensitivity and specificity for the top biomarkers to identify participants with poor physical function who are at elevated risk for mobility disability (SPPB score of $\le$ 7) in both training (AUC = 0.93) and test (AUC = 0.86) data sets.

Notably, a similar collection and rank order of senescence biomarkers were associated with performance on the components of the SPPB score; i.e., 4 m gait speed, chair rise time, and standing balance score. Activin A and ICAM1 were consistently the strongest determinants of all three measures of lower extremity functioning, while six other proteins (MMP7, VEGFA, eotaxin, RANTES, IL15, and PAI1) were consistently identified with two components. Moreover, there was a remarkable similarity in the biomarkers and relative strength of biomarkers predictive of performance between the composite SPPB score and 400 m walk time, a distinct measure of mobility and endurance. Again, activin A, ICAM1, MMP7, VEGFA, and eotaxin emerged as the most robust predictors of both measures. Collectively, these data suggest a common biological pathway of aging, i.e., cellular senescence, may be causally implicated in age-associated declines in physical functioning.

Isometric hand grip strength decline with advancing age is a strong independent predictor of lower extremity strength [30] and recent evidence suggests that grip strength among older adults is predicted by birthweight and pubertal growth, reinforcing its biological underpinnings [31]. Grip strength in females and males, like the SPPB score and 400 m walk time, was most strongly associated with activin A. Somewhat surprisingly, GBM generated a distinct set of top 10 biomarkers for females and males with only ADAMTS13, IL15, and eotaxin being similar. The different biomarkers of the greatest importance for grip strength in women and men did not overlap with the observed differences in the plasma concentrations of several biomarkers between women and men. While the differences in the concentrations of specific biomarkers between women and men are intriguing, we do not have a sound scientific rationale for these observations. In both sexes, there was a marked drop from training to test data sets in the sensitivity and specificity of the biomarkers in identifying persons with weakness. It is possible that the strong effect of age in theses analyses may supersede any predictive value of the selected biomarkers.

In human cross-sectional observational studies, it is challenging to determine causality. On one hand, it is plausible that age-associated increases in circulating biomarkers of systemic senescent cell burden simply parallel age-associated declines in muscle performance and physical function. On the other, evidence in both mouse and human interventional studies supports a mechanistic link. First, young and, to a greater extent, old mice transplanted with senescent preadipocytes exhibited deficits in measures of gait speed, muscle endurance, and muscle strength compared to mice transplanted with non-senescent preadipocytes [10]. Second, mice administered a drug combination to preferentially kill senescent cells (*senolytics*) from 20 to 24 months of age exhibited better maximal gait speed, treadmill endurance, and grip strength relative to mice administered a control intervention [10]. Third, two drugs that suppress the SASP (*senomorphics*), the Jak1/2 inhibitor ruxolitinib and an inhibitor of IKK/NFκB activation, SR12343, have been shown to enhance measures of mobility, strength, and frailty in older and/or progeroid mice [11, 12]. Fourth, limited studies have been conducted in humans, but in a phase 1–2 study of persons with myelofibrosis, ruxolitinib conferred significant improvements in symptom scores in concert with reductions in circulating concentrations of numerous cytokines and, of note, a progressive increase in 6-min walk distance over the course of 6 months [32]. Finally, we have recently identified distinct subpopulations of fibroadipogenic progenitors and postmitotic myofibers that are localized in aging skeletal muscle and express features of cellular senescence [33]. Together, data from these studies and the present study may implicate senescent cells and the SASP in the etiology of age-associated functional decline and generate enthusiasm for the therapeutic potential of emerging senolytic and senomorphic interventions.

The panel of senescence biomarkers used in the current study was developed by identifying candidate SASP proteins in the literature, confirming changes in their expression or abundance in the context of senescence in diverse murine and human cells and tissues, and establishing their detectability and reliable measurement in human blood [13]. Whether the circulating proteins studied herein mediate age-associated changes in physical function or strength was not considered. Even so, activin A, a TGFβ superfamily member that is robustly secreted by senescent preadipocytes and endothelial cells, is a potent negative regulator of skeletal muscle mass. Antibody-based inhibition of circulating activin A results in significant increases in muscle growth and force in mice and lean mass in cynomolgus monkeys [34]. These data support the observed associations and, potentially, a causal role of senescent-cell-derived activin A in age-related declines in physical function and muscle strength.

In contrast, the positive associations of ICAM1 with the composite and all 3 components of the SPPB and 400 m walk time are counterintuitive. ICAM1 is a cell surface glycoprotein expressed on the surface of endothelial cells, fibroblasts, and leukocytes in the immune system. It promotes the adhesion of cells, transendothelial migration of leukocytes to sites of inflammation and, in its soluble form, activation of immune cells [35]. The expression of ICAM1 is triggered by p53 and NFκB signaling and thus, it is a component of the SASP that we previously observed to be secreted by senescent endothelial and epithelial cells and preadipocytes [13]. It is not surprising that circulating levels of ICAM1 have previously been associated with cardiovascular disease onset and progression [36]. Potentially, the plasma concentrations of ICAM1 observed in LIFE study participants do not reflect its biological activity at the cellular level. Indeed, posttranslational modification of soluble ICAM1 by glycosylation directly impacts its ability to adhere and regulate inflammatory events [35]. This feature of ICAM1 was not examined in the present study but may partly account for the observed associations with physical function measures.

## Limitations of the study

The present study has many strengths including the large sample size of older adults with mobility limitations and multi-morbidity, rigorous assessment of physical phenotype, functioning and strength, and an evidence-based and validated platform to measure plasma levels of senescence-associated proteins. However, several limitations need to also be considered. We acknowledge that while there is a scientific foundation for senescent cells to be a plausible source of the circulating biomarkers measured, they are likely not the only source. We note that senescent cells also recruit, anchor, and amplify signals from immune cells, and thus, may indirectly contribute to circulating concentrations of the measured proteins [37]. The cross-sectional nature of these analyses makes the ascertainment of causality challenging. The finite age range of the study participants may limit the generalizability of these findings to older and younger populations. The exclusion of participants with higher levels of physical function and better health status may also make it difficult to dissociate the effects of disease burden from systemic biological age. Finally, participants in the LIFE study were self-selected and/or referred for study enrollment and thus may represent a unique population of older adults.

In summary, we show strong associations between biomarkers of the senescence-associated secretory phenotype, a biologic hallmark of advancing age, and measures of physical functioning and muscle strength in a well-characterized cohort of older adults. Our findings may offer clinical utility in identifying older persons at risk for mobility disability. Considering the notable interest in developing senescent cell-targeting therapies, our data may also be leveraged in clinical research to identify persons who may be most responsive to these interventions and to serve as surrogate endpoints in clinical trials to enhance late-life health and function.

## Supplementary Information

Below is the link to the electronic supplementary material.

Supplementary file1 (DOCX 53 KB)
