# Supplementary material for: Associations between biomarkers of cellular senescence and physical function in humans: observations from the lifestyle interventions for elders (LIFE) study
Source: GeroScience. 2022 Nov 11;44(6):2757–70. doi: 10.1007/s11357-022-00685-2 (PMC9768064; doi:10.1007/s11357-022-00685-2)
Supplement: Supplementary file 1 — Supplementary file1 (DOCX 53 KB) [file 11357_2022_685_MOESM1_ESM.docx]

**SUPPLEMENTAL MATERIALS**

**Supplemental Table 1.** Names and aliases of measured protein biomarkers.

| **Protein name** | **Protein full name** | **Alias** |
| --- | --- | --- |
| Activin A | Activin A | INHBA |
| ADAMTS13 | A disintegrin and metalloproteinase with thrombospondin motifs 13 | VWFCP |
| Eotaxin | Eotaxin | CCL11 |
| Fas | Tumor necrosis factor receptor superfamily member 6 | APT1, TNFRSF6 |
| GDF15 | Growth/differentiation factor 15 | MIC1, NAG1, NRG1 |
| ICAM1 | Intercellular adhesion molecule 1 | CD54 |
| IL6 | Interleukin 6 | IFNB2 |
| IL7 | Interleukin 7 |  |
| IL8 | Interleukin 8 | CXCL8 |
| IL15 | Interleukin 15 |  |
| MCP1 | Monocyte chemotactic protein 1 | CCL2 |
| MDC | Macrophage-derived chemokine | CCL22, SCYA22 |
| MMP1 | Matrix metalloproteinase 1 | Interstitial collagenase |
| MMP2 | Matrix metalloproteinase 2 | CLG4A |
| MMP7 | Matrix metalloproteinase 7 | Matrilysin |
| MMP9 | Matrix metalloproteinase 9 | CLG4B |
| MPO | Myeloperoxidase |  |
| OPN | Osteopontin | SPP1 |
| PAI1 | Plasminogen activator inhibitor 1 | SERPINE1, PLANH1 |
| PARC | Pulmonary and activation-regulated chemokine | CCL18 |
| RAGE | Advanced glycosylation end product-specific receptor |  |
| RANTES | Regulated on Activation, Normal T Cell Expressed and Secreted | CCL5, SCYA5 |
| SOST | Sclerostin | DAND6 |
| TNFα | Tumor necrosis factor alpha | TNFSF2 |
| TNFR1 | Tumor necrosis factor receptor 1 | TNFRSF1A, CD120a |
| TNFR2 | Tumor necrosis factor receptor 2 | TNFRSF1B |
| VEGFA | Vascular endothelial growth factor A | VPF |

**Supplemental Table 2**. Spearman correlations between biomarkers of cellular senescence and chronological age. The Q value column represents the Benjamini and Hochberg false discovery rate.

| **Biomarker** | *r* | P value | Q value |
| --- | --- | --- | --- |
| Activin A | **0.239** | **< 0.001** | **<0.001** |
| ADAMTS13 | **-0.063** | **0.020** | **0.036** |
| Eotaxin | **0.143** | **<0.001** | **<0.001** |
| Fas | **0.138** | **<0.001** | **<0.001** |
| GDF15 | **0.237** | **<0.001** | **<0.001** |
| ICAM1 | 0.003 | 0.912 | 0.912 |
| IL6 | 0.051 | 0.059 | 0.093 |
| IL7 | -0.009 | 0.733 | 0.814 |
| IL8 | 0.046 | 0.087 | 0.130 |
| IL15 | **0.087** | **0.001** | **0.003** |
| MCP1 | -0.010 | 0.710 | 0.814 |
| MDC | -0.005 | 0.843 | 0.875 |
| MMP1 | 0.052 | 0.053 | 0.090 |
| MMP2 | **0.188** | **<0.001** | **<0.001** |
| MMP7 | **0.115** | **<0.001** | **<0.001** |
| MMP9 | -0.044 | 0.103 | 0.146 |
| MPO | 0.011 | 0.685 | 0.814 |
| OPN | **0.128** | **<0.001** | **<0.001** |
| PAI1 | **-0.168** | **<0.001** | **<0.001** |
| PARC | -0.008 | 0.753 | 0.814 |
| RAGE | **0.256** | **<0.001** | **<0.001** |
| RANTES | -0.027 | 0.315 | 0.426 |
| SOST | 0.025 | 0.347 | 0.446 |
| TNFα | **0.083** | **0.002** | **0.004** |
| TNFRI | **0.169** | **<0.001** | **<0.001** |
| TNFRII | **0.099** | **<0.001** | **0.001** |
| VEGFA | **0.140** | **<0.001** | **<0.001** |

**Supplemental Table 3**. Spearman correlations between biomarkers of cellular senescence and Short Physical Performance Battery Score, both unadjusted and adjusted for age, sex, race, and body mass index. The Q value columns represent the Benjamini and Hochberg false discovery rate.

|  | **Unadjusted** | | | **Adjusted** | | |
| --- | --- | --- | --- | --- | --- | --- |
| **Biomarker** | *r* | P value | Q value | *r* | P value | Q value |
| Activin A | **-0.370** | **<0.001** | **<0.001** | **-0.331** | **<0.001** | **<0.001** |
| ADAMTS13 | -0.035 | 0.195 | 0.202 | -0.038 | 0.154 | 0.173 |
| Eotaxin | **-0.185** | **<0.001** | **<0.001** | **-0.174** | **<0.001** | **<0.001** |
| Fas | **-0.145** | **<0.001** | **<0.001** | **-0.112** | **<0.001** | **<0.001** |
| GDF15 | **-0.169** | **<0.001** | **<0.001** | **-0.150** | **<0.001** | **<0.001** |
| ICAM1 | **0.309** | **<0.001** | **<0.001** | **0.305** | **<0.001** | **<0.001** |
| IL6 | **-0.209** | **<0.001** | **<0.001** | **-0.187** | **<0.001** | **<0.001** |
| IL7 | -0.030 | 0.268 | 0.268 | -0.020 | 0.467 | 0.485 |
| IL8 | **-0.075** | **0.005** | **0.008** | **-0.066** | **0.014** | **0.018** |
| IL15 | **-0.279** | **<0.001** | **<0.001** | **-0.269** | **<0.001** | **<0.001** |
| MCP1 | **0.065** | **0.016** | **0.020** | **0.076** | **0.005** | **0.007** |
| MDC | **-0.149** | **<0.001** | **<0.001** | **-0.124** | **<0.001** | **<0.001** |
| MMP1 | -0.053 | 0.051 | 0.059 | -0.040 | 0.137 | 0.161 |
| MMP2 | -0.046 | 0.090 | 0.097 | -0.011 | 0.688 | 0.688 |
| MMP7 | **-0.253** | **<0.001** | **<0.001** | **-0.228** | **<0.001** | **<0.001** |
| MMP9 | **0.075** | **0.005** | **0.008** | **0.079** | **0.004** | **0.005** |
| MPO | -0.051 | 0.056 | 0.063 | -0.027 | 0.314 | 0.339 |
| OPN | **0.077** | **0.004** | **0.007** | **0.088** | **0.001** | **0.002** |
| PAI1 | **0.077** | **0.004** | **0.007** | **0.089** | **0.001** | **0.002** |
| PARC | **0.060** | **0.026** | **0.032** | **0.081** | **0.003** | **0.004** |
| RAGE | **-0.120** | **<0.001** | **<0.001** | **-0.098** | **<0.001** | **<0.001** |
| RANTES | **0.079** | **0.003** | **0.007** | **0.092** | **0.001** | **0.001** |
| SOST | **0.073** | **0.007** | **0.009** | **0.058** | **0.031** | **0.038** |
| TNFα | **-0.108** | **<0.001** | **<0.001** | **-0.086** | **0.001** | **0.002** |
| TNFR1 | **-0.182** | **<0.001** | **<0.001** | **-0.143** | **<0.001** | **<0.001** |
| TNFR2 | **0.073** | **0.007** | **0.009** | **0.102** | **<0.001** | **<0.001** |
| VEGFA | **-0.287** | **<0.001** | **<0.001** | **-0.254** | **<0.001** | **<0.001** |

**Supplemental Table 4**. Spearman correlations between biomarkers of cellular senescence and gait speed, both unadjusted and adjusted for age, sex, race, and body mass index. The Q value columns represent the Benjamini and Hochberg false discovery rate.

|  | **Unadjusted** | | | **Adjusted** | | |
| --- | --- | --- | --- | --- | --- | --- |
| **Biomarker** | *r* | P value | Q value | *r* | Pvalue | Q value |
| Activin A | **-0.167** | **<0.001** | **<0.001** | **-0.131** | **<0.001** | **<0.001** |
| ADAMTS13 | -0.029 | 0.276 | 0.338 | -0.023 | 0.388 | 0.499 |
| Eotaxin | **-0.073** | **0.007** | **0.015** | **-0.077** | **0.004** | **0.010** |
| Fas | **-0.063** | **0.019** | **0.035** | -0.036 | 0.180 | 0.256 |
| GDF15 | **-0.068** | **0.012** | **0.022** | **-0.077** | **0.004** | **0.010** |
| ICAM1 | **0.183** | **<0.001** | **<0.001** | **0.163** | **<0.001** | **<0.001** |
| IL6 | **-0.114** | **<0.001** | **<0.001** | **-0.084** | **0.002** | **0.006** |
| IL7 | **0.082** | **0.002** | **0.007** | **0.103** | **<0.001** | **<0.001** |
| IL8 | **-0.053** | **0.047** | 0.080 | -0.047 | 0.079 | 0.125 |
| IL15 | **-0.112** | **<0.001** | **<0.001** | **-0.105** | **<0.001** | **<0.001** |
| MCP1 | -0.015 | 0.569 | 0.640 | -0.003 | 0.907 | 0.907 |
| MDC | **-0.103** | **<0.001** | **<0.001** | **-0.056** | **0.038** | 0.072 |
| MMP1 | 0.041 | 0.131 | 0.169 | **0.053** | **0.049** | 0.083 |
| MMP2 | -0.001 | 0.985 | 0.985 | 0.018 | 0.513 | 0.622 |
| MMP7 | **-0.194** | **<0.001** | **<0.001** | **-0.174** | **<0.001** | **<0.001** |
| MMP9 | **0.072** | **0.008** | **0.016** | **0.082** | **0.002** | **0.006** |
| MPO | -0.043 | 0.115 | 0.155 | -0.009 | 0.741 | 0.801 |
| OPN | 0.048 | 0.078 | 0.111 | 0.035 | 0.199 | 0.268 |
| PAI1 | **0.079** | **0.004** | **0.010** | **0.125** | **<0.001** | **<0.001** |
| PARC | 0.026 | 0.329 | 0.386 | **0.055** | **0.043** | 0.077 |
| RAGE | 0.008 | 0.781 | 0.843 | 0.004 | 0.882 | 0.907 |
| RANTES | **0.085** | **0.002** | **0.006** | **0.110** | **<0.001** | **<0.001** |
| SOST | 0.051 | 0.056 | 0.090 | 0.017 | 0.536 | 0.622 |
| TNFα | -0.005 | 0.848 | 0.881 | 0.016 | 0.553 | 0.622 |
| TNFR1 | **-0.078** | **0.004** | **0.010** | -0.045 | 0.092 | 0.138 |
| TNFR2 | 0.048 | 0.073 | 0.110 | **0.065** | **0.016** | **0.032** |
| VEGFA | **-0.119** | **<0.001** | **<0.001** | **-0.083** | **0.002** | **0.006** |

**Supplemental Table 5**. Spearman correlations between biomarkers of cellular senescence and repeated chair rise time, both unadjusted and adjusted for age, sex, race, and body mass index. The Q value columns represent the Benjamini and Hochberg false discovery rate.

|  | **Unadjusted** | | | **Adjusted** | | |
| --- | --- | --- | --- | --- | --- | --- |
| **Biomarker** | *r* | P value | Q value | *r* | Pvalue | Q value |
| Activin A | **0.163** | **<0.001** | **<0.001** | **0.175** | **<0.001** | **<0.001** |
| ADAMTS13 | -0.008 | 0.754 | 0.754 | -0.008 | 0.765 | 0.765 |
| Eotaxin | **0.108** | **<0.001** | **<0.001** | **0.116** | **<0.001** | **<0.001** |
| Fas | 0.028 | 0.296 | 0.366 | 0.036 | 0.188 | 0.242 |
| GDF15 | 0.022 | 0.419 | 0.471 | 0.038 | 0.159 | 0.215 |
| ICAM1 | **-0.188** | **<0.001** | **<0.001** | **-0.184** | **<0.001** | **<0.001** |
| IL6 | **0.099** | **<0.001** | **0.001** | **0.109** | **<0.001** | **<0.001** |
| IL7 | **0.088** | **0.001** | **0.004** | **0.087** | **0.001** | **0.005** |
| IL8 | 0.047 | 0.079 | 0.112 | 0.048 | 0.074 | 0.105 |
| IL15 | **0.144** | **<0.001** | **<0.001** | **0.147** | **<0.001** | **<0.001** |
| MCP1 | **-0.075** | **0.006** | **0.017** | **-0.073** | **0.007** | **0.016** |
| MDC | **0.064** | **0.017** | **0.037** | **0.061** | **0.024** | **0.042** |
| MMP1 | **0.064** | **0.018** | **0.037** | **0.065** | **0.016** | **0.034** |
| MMP2 | -0.028 | 0.306 | 0.366 | -0.024 | 0.378 | 0.425 |
| MMP7 | **0.053** | **0.050** | 0.079 | **0.056** | **0.038** | 0.064 |
| MMP9 | -0.052 | 0.053 | 0.079 | -0.052 | 0.053 | 0.079 |
| MPO | 0.027 | 0.312 | 0.366 | 0.029 | 0.281 | 0.330 |
| OPN | -0.040 | 0.136 | 0.184 | -0.035 | 0.198 | 0.243 |
| PAI1 | -0.015 | 0.581 | 0.603 | -0.020 | 0.449 | 0.484 |
| PARC | **-0.063** | **0.019** | **0.038** | **-0.063** | **0.020** | **0.039** |
| RAGE | **0.071** | **0.008** | **0.022** | **0.079** | **0.003** | **0.010** |
| RANTES | -0.015 | 0.571 | 0.603 | -0.019 | 0.475 | 0.493 |
| SOST | **-0.062** | **0.021** | **0.038** | **-0.054** | **0.045** | 0.071 |
| TNFα | **0.061** | **0.024** | **0.040** | **0.066** | **0.014** | **0.032** |
| TNFR1 | **0.067** | **0.012** | **0.031** | **0.081** | **0.003** | **0.009** |
| TNFR2 | **-0.079** | **0.003** | **0.011** | **-0.074** | **0.006** | **0.016** |
| VEGFA | **0.150** | **<0.001** | **<0.001** | **0.160** | **<0.001** | **<0.001** |

**Supplemental Table 6**. Spearman correlations between biomarkers of cellular senescence and standing balance score, both unadjusted and adjusted for age, sex, race, and body mass index. The Q value columns represent the Benjamini and Hochberg false discovery rate.

|  | **Unadjusted** | | | **Adjusted** | | |
| --- | --- | --- | --- | --- | --- | --- |
| **Biomarker** | *r* | P value | Q value | *r* | Pvalue | Q value |
| Activin A | **-0.214** | **<0.001** | **<0.001** | **-0.167** | **<0.001** | **<0.001** |
| ADAMTS13 | -0.020 | 0.448 | 0.551 | -0.029 | 0.276 | 0.373 |
| Eotaxin | **-0.096** | **<0.001** | **<0.001** | **-0.067** | **0.012** | **0.034** |
| Fas | **-0.121** | **<0.001** | **<0.001** | **-0.086** | **0.001** | **0.004** |
| GDF15 | **-0.175** | **<0.001** | **<0.001** | **-0.129** | **<0.001** | **<0.001** |
| ICAM1 | **0.126** | **<0.001** | **<0.001** | **0.132** | **<0.001** | **<0.001** |
| IL6 | **-0.119** | **<0.001** | **<0.001** | **-0.100** | **<0.001** | **<0.001** |
| IL7 | -0.015 | 0.576 | 0.648 | -0.014 | 0.603 | 0.679 |
| IL8 | -0.039 | 0.145 | 0.230 | -0.029 | 0.275 | 0.373 |
| IL15 | **-0.171** | **<0.001** | **<0.001** | **-0.156** | **<0.001** | **<0.001** |
| MCP1 | 0.025 | 0.363 | 0.515 | 0.029 | 0.275 | 0.373 |
| MDC | **-0.065** | **0.016** | **0.032** | **-0.062** | **0.022** | 0.053 |
| MMP1 | **-0.060** | **0.027** | **0.048** | -0.048 | 0.073 | 0.151 |
| MMP2 | **-0.077** | **0.004** | **0.009** | -0.038 | 0.163 | 0.275 |
| MMP7 | **-0.152** | **<0.001** | **<0.001** | **-0.127** | **<0.001** | **<0.001** |
| MMP9 | 0.010 | 0.706 | 0.733 | 0.006 | 0.837 | 0.837 |
| MPO | -0.001 | 0.998 | 0.988 | 0.013 | 0.633 | 0.684 |
| OPN | 0.015 | 0.566 | 0.648 | 0.042 | 0.119 | 0.213 |
| PAI1 | 0.040 | 0.138 | 0.230 | 0.018 | 0.515 | 0.605 |
| PARC | 0.013 | 0.639 | 0.639 | 0.020 | 0.451 | 0.554 |
| RAGE | **-0.097** | **<0.001** | **<0.001** | **-0.053** | **0.049** | 0.110 |
| RANTES | 0.030 | 0.269 | 0.404 | 0.027 | 0.321 | 0.413 |
| SOST | 0.024 | 0.383 | 0.517 | 0.032 | 0.237 | 0.373 |
| TNFα | **-0.068** | **0.012** | 0.025 | -0.044 | 0.101 | 0.195 |
| TNFR1 | **-0.155** | **<0.001** | **<0.001** | **-0.113** | **<0.001** | **<0.001** |
| TNFR2 | -0.020 | 0.449 | 0.551 | 0.009 | 0.743 | 0.771 |
| VEGFA | **-0.190** | **<0.001** | **<0.001** | **-0.157** | **<0.001** | **<0.001** |

**Supplemental Table 7**. Spearman correlations between biomarkers of cellular senescence and 400m walk time, both unadjusted and adjusted for age, sex, race, and body mass index. The Q value columns represent the Benjamini and Hochberg false discovery rate.

|  | **Unadjusted** | | | **Adjusted** | | |
| --- | --- | --- | --- | --- | --- | --- |
| **Biomarker** | *r* | P value | Q value | *r* | Pvalue | Q value |
| Activin A | **0.239** | **<0.001** | **<0.001** | **0.182** | **<0.001** | **<0.001** |
| ADAMTS13 | -0.007 | 0.801 | 0.832 | -0.019 | 0.492 | 0.532 |
| Eotaxin | **0.149** | **<0.001** | **<0.001** | **0.155** | **<0.001** | **<0.001** |
| Fas | **0.131** | **<0.001** | **<0.001** | **0.086** | **0.001** | **0.003** |
| GDF15 | **0.146** | **<0.001** | **<0.001** | **0.149** | **<0.001** | **<0.001** |
| ICAM1 | **-0.140** | **<0.001** | **<0.001** | **-0.116** | **<0.001** | **<0.001** |
| IL6 | **0.185** | **<0.001** | **<0.001** | **0.139** | **<0.001** | **<0.001** |
| IL7 | 0.032 | 0.234 | 0.275 | 0.007 | 0.799 | 0.830 |
| IL8 | **0.082** | **0.002** | **0.004** | **0.073** | **0.006** | **0.015** |
| IL15 | **0.116** | **<0.001** | **<0.001** | **0.104** | **<0.001** | **<0.001** |
| MCP1 | 0.047 | 0.084 | 0.120 | 0.027 | 0.310 | 0.364 |
| MDC | **0.151** | **<0.001** | **<0.001** | **0.088** | **0.001** | **0.003** |
| MMP1 | **0.080** | **0.003** | **0.005** | **0.067** | **0.013** | **0.027** |
| MMP2 | **0.071** | **0.008** | **0.012** | 0.042 | 0.119 | 0.169 |
| MMP7 | **0.206** | **<0.001** | **<0.001** | **0.175** | **<0.001** | **<0.001** |
| MMP9 | -0.020 | 0.460 | 0.460 | -0.035 | 0.193 | 0.248 |
| MPO | **0.103** | **<0.001** | **<0.001** | **0.053** | **0.048** | 0.076 |
| OPN | 0.041 | 0.125 | 0.161 | **0.060** | **0.026** | **0.044** |
| PAI1 | 0.031 | 0.258 | 0.290 | -0.023 | 0.398 | 0.448 |
| PARC | 0.037 | 0.167 | 0.205 | -0.004 | 0.872 | 0.872 |
| RAGE | 0.044 | 0.104 | 0.141 | 0.048 | 0.075 | 0.113 |
| RANTES | -0.004 | 0.882 | 0.882 | -0.034 | 0.211 | 0.259 |
| SOST | **-0.078** | **0.004** | **0.006** | -0.038 | 0.159 | 0.215 |
| TNFα | **0.095** | **<0.001** | **<0.001** | **0.062** | **0.021** | **0.041** |
| TNFR1 | **0.198** | **<0.001** | **<0.001** | **0.147** | **<0.001** | **<0.001** |
| TNFR2 | **0.087** | **0.001** | **0.003** | **0.061** | **0.025** | **0.044** |
| VEGFA | **0.227** | **<0.001** | **<0.001** | **0.175** | **<0.001** | **<0.001** |

**Supplemental Table 8**. Spearman correlations between biomarkers of cellular senescence and grip strength in females, both unadjusted and adjusted for age, race, and body mass index. The Q value columns represent the Benjamini and Hochberg false discovery rate.

|  | **Unadjusted** | | | **Adjusted** | | |
| --- | --- | --- | --- | --- | --- | --- |
| **Biomarker** | *r* | P value | Q value | *r* | Pvalue | Q value |
| Activin A | **-0.157** | **<0.001** | **<0.001** | **-0.104** | **0.002** | **0.016** |
| ADAMTS13 | **0.075** | **0.028** | 0.076 | 0.048 | 0.159 | 0.358 |
| Eotaxin | **-0.075** | **0.028** | 0.076 | -0.047 | 0.173 | 0.360 |
| Fas | **-0.090** | **0.009** | **0.029** | -0.063 | 0.065 | 0.194 |
| GDF15 | **-0.148** | **<0.001** | **<0.001** | **-0.092** | **0.007** | **0.033** |
| ICAM1 | -0.049 | 0.156 | 0.267 | -0.059 | 0.084 | 0.227 |
| IL6 | -0.064 | 0.061 | 0.151 | **-0.083** | **0.015** | 0.055 |
| IL7 | -0.002 | 0.965 | 0.996 | -0.005 | 0.888 | 0.888 |
| IL8 | 0.014 | 0.678 | 0.732 | 0.031 | 0.368 | 0.552 |
| IL15 | -0.039 | 0.254 | 0.376 | -0.015 | 0.672 | 0.816 |
| MCP1 | -0.030 | 0.389 | 0.526 | -0.043 | 0.215 | 0.415 |
| MDC | 0.024 | 0.476 | 0.612 | 0.006 | 0.852 | 0.884 |
| MMP1 | -0.049 | 0.155 | 0.267 | -0.040 | 0.240 | 0.428 |
| MMP2 | **-0.101** | **0.003** | **0.012** | -0.056 | 0.101 | 0.249 |
| MMP7 | -0.043 | 0.212 | 0.336 | -0.017 | 0.628 | 0.816 |
| MMP9 | 0.018 | 0.610 | 0.686 | 0.012 | 0.726 | 0.816 |
| MPO | 0.0001 | 0.996 | 0.996 | -0.013 | 0.702 | 0.816 |
| OPN | -0.048 | 0.158 | 0.267 | -0.017 | 0.611 | 0.816 |
| PAI1 | 0.038 | 0.265 | 0.376 | -0.012 | 0.717 | 0.816 |
| PARC | -0.059 | 0.083 | 0.188 | **-0.083** | **0.016** | 0.055 |
| RAGE | **-0.179** | **<0.001** | **<0.001** | **-0.106** | **0.002** | **0.016** |
| RANTES | 0.018 | 0.610 | 0.686 | 0.009 | 0.784 | 0.847 |
| SOST | 0.022 | 0.520 | 0.638 | 0.039 | 0.253 | 0.428 |
| TNFα | **-0.106** | **0.002** | **0.009** | **-0.096** | **0.005** | **0.027** |
| TNFR1 | **-0.135** | **<0.001** | **0.001** | **-0.120** | **<0.001** | **0.006** |
| TNFR2 | **-0.129** | **<0.001** | **0.001** | **-0.124** | **<0.001** | **0.006** |
| VEGFA | -0.056 | 0.105 | 0.218 | -0.033 | 0.334 | 0.531 |

**Supplemental Table 9**. Spearman correlations between biomarkers of cellular senescence and grip strength in males, both unadjusted and adjusted for age, race, and body mass index. The Q value columns represent the Benjamini and Hochberg false discovery rate.

|  | **Unadjusted** | | | **Adjusted** | | |
| --- | --- | --- | --- | --- | --- | --- |
| **Biomarker** | *r* | P value | Q value | *r* | Pvalue | Q value |
| Activin A | **-0.160** | **0.001** | **0.009** | **-0.120** | **0.011** | 0.104 |
| ADAMTS13 | -0.024 | 0.609 | 0.685 | -0.049 | 0.303 | 0.584 |
| Eotaxin | **-0.115** | **0.014** | 0.070 | -0.062 | 0.192 | 0.533 |
| Fas | -0.042 | 0.376 | 0.483 | -0.026 | 0.576 | 0.741 |
| GDF15 | **-0.114** | **0.015** | 0.070 | -0.058 | 0.218 | 0.533 |
| ICAM1 | -0.014 | 0.763 | 0.824 | -0.002 | 0.973 | 0.973 |
| IL6 | **-0.124** | **0.008** | 0.056 | **-0.119** | **0.012** | 0.104 |
| IL7 | -0.005 | 0.913 | 0.948 | -0.007 | 0.881 | 0.927 |
| IL8 | -0.002 | 0.968 | 0.968 | 0.006 | 0.893 | 0.927 |
| IL15 | -0.072 | 0.126 | 0.261 | -0.060 | 0.204 | 0.533 |
| MCP1 | 0.050 | 0.290 | 0.411 | 0.042 | 0.372 | 0.627 |
| MDC | -0.058 | 0.216 | 0.364 | -0.047 | 0.324 | 0.584 |
| MMP1 | -0.088 | 0.064 | 0.215 | -0.062 | 0.190 | 0.533 |
| MMP2 | **-0.149** | **0.002** | **0.014** | **-0.098** | **0.039** | **0.263** |
| MMP7 | -0.056 | 0.237 | 0.376 | -0.037 | 0.432 | 0.649 |
| MMP9 | **0.094** | **0.047** | 0.181 | 0.058 | 0.223 | 0.533 |
| MPO | 0.039 | 0.412 | 0.505 | 0.029 | 0.542 | 0.732 |
| OPN | -0.046 | 0.328 | 0.443 | 0.016 | 0.734 | 0.901 |
| PAI1 | 0.085 | 0.072 | 0.215 | 0.010 | 0.839 | 0.927 |
| PARC | -0.053 | 0.261 | 0.391 | -0.056 | 0.237 | 0.533 |
| RAGE | -0.078 | 0.098 | 0.222 | 0.008 | 0.862 | 0.927 |
| RANTES | 0.027 | 0.566 | 0.664 | 0.037 | 0.429 | 0.649 |
| SOST | **0.221** | **<0.001** | **<0.001** | **0.218** | **<0.001** | **<0.001** |
| TNFα | -0.066 | 0.160 | 0.290 | -0.068 | 0.154 | 0.533 |
| TNFR1 | -0.066 | 0.161 | 0.290 | -0.034 | 0.474 | 0.674 |
| TNFR2 | -0.078 | 0.099 | 0.222 | -0.047 | 0.324 | 0.584 |
| VEGFA | -0.081 | 0.088 | 0.222 | -0.060 | 0.207 | 0.533 |

**Supplemental Table 10**. Assay performance characteristics, including measurement range, sensitivity, and the coefficient of variation (CV) for biomarkers of cellular senescence.

| **Biomarker** | *Range (pg/ml)** | *Sensitivity (pg/ml)* | *CV(%)* |
| --- | --- | --- | --- |
| Activin A | 15.6-1000 | 7.85 | 11.36 |
| ADAMTS13 | 16000-3900000 | 1270 | 11.39 |
| Eotaxin | 32.3-23580 | 1.81 | 10.58 |
| Fas | 144-35000 | 3.2 | 12.29 |
| GDF15 | 18.5-4500 | 1.2 | 15.41 |
| ICAM1 | 7000-1700000 | 87.9 | 12.50 |
| IL6 | 0.7-2800 | 0.135 | 12.62 |
| IL7 | 0.9-3620 | 0.140 | 18.14 |
| IL8 | 0.879-3600 | 0.07 | 8.83 |
| IL15 | 0.6-2350 | 0.167 | 24.50 |
| MCP1 | 4.2-3070 | 0.6 | 7.48 |
| MDC | 53.5-13000 | 8.5 | 9.82 |
| MMP1 | 49.4-12000 | 2.7 | 13.86 |
| MMP2 | 276-67000^*^ | 108 | 11.14 |
| MMP7 | 226-55000 | 23.2 | 14.26 |
| MMP9 | 123-30000^*^ | 13.6 | 8.63 |
| MPO | 123-30000^*^ | 26.2 | 13.98 |
| OPN | 1440-350000 | 413 | 22.21 |
| PAI1 | 18.1-4400^*^ | 0.7 | 13.60 |
| PARC | 18.5-4500^*^ | 0.3 | 10.83 |
| RAGE | 123-30000 | 7.2 | 13.56 |
| RANTES | 20.6-5000^*^ | 1.8 | 20.11 |
| SOST | 10.3-2500 | 7 | 18.84 |
| TNFα | .757-3100 | 0.54 | 9.93 |
| TNFR1 | 49.4-12000 | 41 | 15.89 |
| TNFR2 | 11.1-2700 | 0.5 | 7.44 |
| VEGFA | 7-4280 | 1.17 | 6.81 |

^*^ Denotes biomarkers analyzed in plasma samples at 1:100 dilution. All other biomarkers analyzed in plasma samples at 1:2 dilution.
